# Genealogy

**Published:** 1914-03

**Authors:** 


### GENEALOGY

We started to abstract an article as to whether cancer was truly a hereditary disease, but we found in it, merely a citation of the old theory that cancer occurs with undue frequence in the residuary members of tuberculous families, that certain very incomplete statistics of two families showed an incidence of cancer somewhere near what would be expected by the Mendelian law, and some opinions. This problem as well as many others can be decided positively only by actual evidence and such evidence, except in a very limited heredity, must depend upon accurate genealogic study. It is of course impossible to obtain accurate pathologic data from the distant past, especially from ordinary family records but in regard to cancer, tuberculosis, gout and a few other diseases, some degree of reliance may be placed upon even such records. More general problems of natural fecundity, longevity, stature, etc., might be elucidated to a still greater degree. We feel that genealogy might well be taken up by some members of our own profession—Dr. Stiles's work, for example, is a model, though not conspicuously valuable from the medical standpoint—and that our profession ought to influence professional genealogists so that they may realize that there are vital interests connected with their study, of much greater value than trite eulogies, and eligibility to various organizations.

